# Empirically derived dietary patterns and obesity among Iranian Adults: Yazd Health Study‐TAMYZ and Shahedieh cohort study

**DOI:** 10.1002/fsn3.1538

**Published:** 2020-03-31

**Authors:** Sahar Sarkhosh‐Khorasani, Hassan Mozaffari‐Khosravi, Masoud Mirzaei, Azadeh Nadjarzadeh, Mahdieh Hosseinzadeh

**Affiliations:** ^1^ Nutrition and Food Security Research Center Shahid Sadoughi University of Medical Sciences Yazd Iran; ^2^ Department of Nutrition School of Public Health Shahid Sadoughi University of Medical Sciences Yazd Iran; ^3^ Yazd Cardiovascular Research Centre Shahid Sadoughi University of Medical Sciences Yazd Iran

**Keywords:** adults, diet, Iran, obesity

## Abstract

The aim was to determine the relationship between dietary patterns derived by principal component analysis (PCA) in association with obesity from a large group of Iranian adults in the urban and suburb areas. A cross‐sectional study was conducted on 10,693 Iranian adults. The data were collected from two cohort studies: Shahedieh city annexed to Yazd area as well as Yazd Health Study (YaHS)‐TAMYZ (Yazd Nutrition Survey in Persian) in urban area. Dietary intakes were assessed using a validated semi‐quantitative food frequency questionnaire. The PCA was applied to identify the dietary patterns. Multiple logistic regressions were run to assess the relationship between dietary patterns and obesity. In Shahedieh cohort study, three major dietary patterns were identified traditional, unhealthy, and prudent pattern. Prudent pattern was associated with lower odds of obesity (OR: 0.68; 95% CI: 0.53, 0.88). Higher adherence to the unhealthy (OR: 1.24; 95% CI: 1.02, 1.50) and traditional (OR: 1.38; 95% CI: 1.11, 1.72) patterns was related to greater odds of obesity. Moreover, we identified traditional and unhealthy dietary patterns in YaHS study. Higher adherence to the unhealthy dietary pattern was associated with greater odds of obesity (OR: 1.21 95% CI: 1.02, 1.44). Greater adherence to unhealthy dietary patterns was associated with higher odds of obesity in participants. Greater adherence to traditional and prudent dietary patterns increased and decreased the obesity odds, respectively. Further prospective studies are needed to find out the causal relationship between the variables.

## Graphical abstract

This paper aimed to evaluate the relationship between major dietary patterns derived from posteriori methods and obesity among a large sample of both suburb and rural Iranian adults.

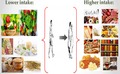

## 1 INTRODUCTION

Various factors including behavior, environment, heredity, socioeconomic status (SES), and culture can lead to obesity (Heymsfield & Wadden, 2017). The prevalence of overweight and obesity has a growing trend throughout the world (Roberto et al., 2015). Since 1980, the prevalence of obesity has doubled in more than 70 countries and is increasing continuously in most other countries (GBD, 2015 Obesity Collaborators, 2017). According to the statistics, a total of 603.7 million adults were obese in 2015 (GBD, 2015 Obesity Collaborators, 2017). The increasing prevalence of obesity increased the prevalence of co‐morbidities related to obesity around the world (GBD, 2015 Obesity Collaborators, 2017). Progressive weight gain may cause a variety of co‐morbidities, such as type 2 diabetes, hypertension, dyslipidemia, cardiovascular disease, liver dysfunction, respiratory and musculoskeletal disorders, subfertility, psychosocial problems, and certain types of cancer (Kyrou, Randeva, Tsigos, Kaltsas, & Weickert, 2018). As a result, the morality rate increases (Varraso et al., 2012).

Diet, as an important lifestyle factor, is associated with body composition that can prevent obesity (Baynes, 1991; Cho, Dietrich, Brown, Clark, & Block, 2003; Lin, Huang, & French, 2004). Research over the association between diet and obesity has mostly dealt with nutrients (García et al., 2012; Huang et al., 2011; Saneei, Salehi‐Abargouei, & Esmaillzadeh, 2013; Westerterp‐Plantenga, Nieuwenhuizen, Tome, Soenen, & Westerterp, 2009; Zhou, Li, Zhou, Sun, & Liu, 2010), foods (Abete, Parra, Crujeiras, Goyenechea, & Martinez, 2008; Cope, Erdman, & Allison, 2008; Juul & Hemmingsson, 2015; Nkondjock & Receveur, 2003), and food groups (Crujeiras, Parra, Rodríguez, de Morentin, & Martínez, 2006; Freisling et al., 2018; Liu et al., 2003; Messina, 1999; Zemel et al., 2005). Dietary pattern was developed as a new comprehensive approach toward the relationship between diet and diseases (Hu, 2002) by considering the complex interactions between nutrients and foods (van Dam, 2005; Jacka et al., 2010). In order to extract the dietary patterns, two approaches have been applied to combine nutrients or foods (Newby & Tucker, 2004). The first is a priori or theoretical approach including the definition of certain scores or indices as well as an empirical or posteriori definition based on the statistical measurements (Román‐Vinas et al., 2009).

The studies over the association between dietary patterns and obesity using the posteriori methods concluded that higher intakes of high‐fiber cereal, fruits, and vegetables products were inversely associated with obesity (Huybrechts et al., 2017; Livingstone & McNaughton, 2017; Papavagelis et al., 2018; Shaker‐Hosseini & Ghodrati, 2017; Shu et al., 2015; Slagter et al., 2018; Zhang et al., 2015). However, odds of obesity increased in dietary patterns with greater intakes of red meat, refined grains, and carbohydrates (Huybrechts et al., 2017; Livingstone & McNaughton, 2017; Papavagelis et al., 2018; Shaker‐Hosseini & Ghodrati, 2017; Shu et al., 2015; Slagter et al., 2018; Zhang et al., 2015). In this regard, a cross‐sectional study investigated the association between traditional dietary pattern and obesity among Chinese adults (Shu et al., 2015). According to the results, traditional dietary pattern, consisting of high amounts of fruits, vegetables, seeds, and tubers was associated with lower odds of obesity (Shu et al., 2015). On the contrary, animal dietary pattern that included high amounts of rice, red meat, and fat was associated with higher odds of obesity (Shu et al., 2015). Furthermore, adherence to the western/unhealthy dietary pattern was related to increased odds of obesity, whereas a prudent/healthy dietary pattern decreased the odds of obesity among the urban Iranian women (Rezazadeh & Rashidkhani, 2010; Shaker‐Hosseini & Ghodrati, 2017). In this regard, similar results were reported in adults from Canada (Paradis, Godin, Perusse, & Vohl, 2009) and Argentina (Pou, del Pilar Díaz, De La Quintana, Forte, & Aballay, 2016).

According to a research over 52 countries from eight different geographical regions, mean body mass index (BMI) of people from the Middle East countries (27.4) was higher than individuals from Europe (26.7), South America (26.7), Africa (26.7), China (24.4), Australia (27.0), and other parts of Asia (24.9; Yusuf et al., 2005). However, mean BMI of adults from the Middle Eastern countries (27.4) was lower than North America (27.7; Yusuf et al., 2005).

This study was conducted considering the high prevalence of obesity (Asfaw, 2007) as well as different cultural and socioeconomic levels among the Middle Eastern populations (Monteiro, Moura, Conde, & Popkin, 2004). Our aim was to investigate the association between dietary patterns and obesity in Iran, where the existing various dietary patterns may provide some novel insights into the diet–disease relationships. To the best of our knowledge, the association between principal component analysis (PCA)‐derived dietary patterns and obesity was studied in few researches with low sample size in Middle East countries (Jomaa et al., 2016; Mirzababaei et al., 2019; Naja et al., 2011; Rezazadeh & Rashidkhani, 2010; Shaker‐Hosseini & Ghodrati, 2017). Moreover, most of these studies were conducted in urban areas, where the culture and dietary habits are different from the suburb regions (Hooper, Calvert, Thompson, Deetlefs, & Burney, 2008; Maruapula & Chapman‐Novakofski, 2007). This study targeted at evaluating the relationship between major dietary patterns derived from posteriori methods and obesity among a large sample of Iranian adults living in urban and suburb areas.

## 2 MATERIAL AND METHODS

### 2.1 Study design and population

The present cross‐sectional study was carried out based on the data collected from two cohort studies (Shahedieh and YaHS). Dietary foods and supplements have been addressed in our substudy, called Yazd Nutrition Survey (YNS), locally known as TAMYZ in Persian. This component of YaHS involved administration of a food frequency questionnaire (FFQ) consisting of 178 food items and 551 questions (Esfahani, Asghari, Mirmiran, & Azizi, 2010). All participants of YaHS were included in TAMIZ, which was started in December 2015 (Mirzaei, Salehi‐Abargouei, Mirzaei, & Mohsenpour, 2017). Detailed information about the design and baseline population of YaHS study was published previously (Mirzaei et al., 2017). This research included a total of 8,966 individuals from the suburb region (Shahedieh) aged 35–70 years and 10,038 people from the urban and rural areas (YaHS study) aged 20–70 years in Iran. Considering the above‐mentioned substudies, the exclusion criteria were being on a weight loss or specific diet and having a history of diseases such as diabetes, cardiovascular diseases, stroke, fatty liver, hypertension, cancer, and thyroid, since such diseases may change the participants' diet. Furthermore, participants with a total daily energy intake of less than 800 or higher than 6,500 kcal were excluded.

### 2.2 Dietary assessment

The semi‐quantitative FFQ was administered to assess the dietary foods and supplements. The original semi‐quantitative FFQ contains 168 items, but 10 more questions were added on consumption of Yazd‐specific frequently consumed food items, which made a total of 178 food items. The semi‐quantitative FFQ was previously validated for the Iranian population (Esfahani et al., 2010), so the questionnaire was completed by trained interviewers. Participants were supposed to report the amount and frequency of consuming each food item per month, week, or day in the past year. Moreover, a food photograph book was used for all participants as a reference, so that they could estimate the portion size of foods as a unit accurately. Participants were also asked to report their intake frequency with regard to all food items based on 10 multiple‐choice frequency response categories ranging from “never or less than once a month” to “10 or more times per day.” Later, the amount of food consumed at each intake was estimated using questions with five predefined answers.

### 2.3 Anthropometric assessment

Participants' body weight was measured in standing position with light clothing. All anthropometric indices were measured three times: before the interview, after completing one‐third of the questionnaire, and after completing two‐thirds of the questions. Participants' height was also measured to the nearest centimeter with barefoot while their heads, shoulder blades, buttocks, and heels were rested against the wall. BMI (kg/m^2^) was calculated using weight and height measurements according to the following formula: weight (kg)/height squared (m^2^). Waist circumference was recorded to the nearest 0.5 cm using nonstretch tape placed midway between iliac crest and lowest rib while participants were in the standing position (Edwards, Williams‐Roberts, & Sahely, 2008).

### 2.4 Assessment of covariates

The demographic and medical history questionnaires were also administered, and the related information was collected from all participants: age, gender, marital status, tobacco smoking, SES, and diseases. The SES score was calculated to determine the individuals' SES based on the infrastructure facilities (source of drinking water and sanitation facility), housing condition (e.g., the number of rooms, type of home ownership), durable assets' ownership (e.g., dishwasher, car, television), and education level (Karyani et al., 2019). Later, the total SES score, ranging from 0 to 3, was calculated by summing up the assigned scores; a score of 3 showed high SES. In addition, the Iranian version of International Physical Activity Questionnaire was applied to calculate the participants' physical activity (Moghaddam et al., 2012) and individuals with more than 1 hr of activity per week were considered as physically active.

### 2.5 Statistical analysis

To determine the major dietary patterns based on the food groups (*N* = 22), the PCA was applied and the factors were rotated using the varimax rotation. Furthermore, the study factors were naturally interpreted in conjunction with eigenvalues >1.5 and the scree plot was determined. The derived dietary patterns were labeled according to data interpretation and similar studies. To calculate the factor score of each identified pattern, the food group intakes weighted by their factor loadings were summed for each participant. Later, the participants were categorized based on the dietary pattern scores' quartiles (quartile 1: low consumption, quartile 4: high consumption of a given food pattern). Next, the participants' characteristics were measured across quartiles of each dietary pattern and the data were calculated by mean ± standard deviation for continuous variables and percentage for categorical variables. Analysis of variance was run to describe the mean differences of the continuous variables, and the chi‐squared test was applied to determine the difference between categorical variables. Multivariable logistic regression analysis was also used to study the association of dietary patterns with obesity in different models. Initially, the confounder variables were adjusted: age, energy intake (kcal/d), gender, smoking status (nonsmoker, ex‐smoker, current smoker), SES (weak, moderate, high), marital status (married, single, widowed, divorced), physical activity level (never, <1, >1 hr/week), and diseases. With regard to all analyses, we considered the first quartiles of dietary pattern scores as the reference. The quartile categories were also considered as ordinal variables in the analyses to calculate the overall trend of odds ratios (OR) across increasing quartiles of dietary pattern scores. The IBM SPSS version 20.0 was run to analyze the data, and the significant *p* value was set at <.05.

## 3 RESULT

### 3.1 Study population characteristic

In Shahedieh cohort study, 73.3% of the participants were in the age range of 35–49 years and 26.6% were above 50 years. Prevalence of obesity was calculated as 26.7% (men, 10.1%; women, 16.6%). Food groups and their corresponding food items used in PCA to derive dietary patterns are shown in Table 1. The results of factor analysis showed three dietary patterns: “Traditional” (highly loaded by vegetable, red and processed meats, fish, soft drinks, fruits, nuts, pickles, eggs, legumes, dairy, mayonnaise, potatoes, refined grains, snacks, poultry, vegetable oils, sweet and sugars, olive group and tea and coffee), “Unhealthy” (highly loaded by sweet and sugars, tea and coffee, eggs, potatoes, and snack but low intake of vegetables, fruits, olive group, and dairy), and "Prudent" (highly loaded by fruits, vegetables, whole grains, dairy, but low intake of pizza, snacks, soft drinks, refined grains, and vegetable oils). The factor loading matrixes for these dietary patterns are shown in Table 2. These dietary patterns explained 28.1% of the whole variance. The participants' characteristics and dietary intakes across dietary patterns' quartiles are represented in Tables S1 and S3, respectively. Individuals in the highest quartiles of all dietary patterns were significantly more likely to be males and married, but they had a higher intake of energy, red and processed meat, vegetable, fruits, egg, potato, refined grains, mayonnaise, and nuts compared to those in the lowest quartiles. Participants in the top quartile of traditional pattern were younger and had higher weight and height compared with those in the lowest quartile. Among the people with unhealthy dietary pattern, the highest quartile members were likely to be older and had higher weight, height, and SES but, they had lower physical activity compared with individuals in the lowest quartile. Furthermore, members of the highest quartile of the prudent pattern had a lower prevalence of central obesity, BMI, and SES but they are more likely to be younger compared with those in the lowest quartile.

**Table 1:** Food groups and their corresponding food items used in principal component analysis to derive dietary patterns

Food groups	Food items
Red & processed meats	Sausages; Hamburgers; Lamb; Beef; Kebab; Beef liver; lamb organ (Tongue; Tripe; Head and trotters; Brain; Foot; Abomasum)
Fish	Canned fish; other fish
Poultry	Chicken with or without skin (Liver; Heart; Gizzard)
Eggs	Eggs
Dairy	Cheese; Curd; Ice‐cream; Flavored; chocolate; coffee and honey milk; Cream; Dough
Fruits	Pears; Apricots; Cherries; Apples; Grapes; Bananas; Cantaloupe; Melons; Watermelon; Kiwi; Strawberries; Peaches; Mulberry; Plums; Persimmons; Pomegranates; Figs; Dates; Greengage; Sour cherry; Citrus fruits; All types of canned fruit; Dried Figs; Dried Mulberries; Raisins; dried plums; dried apricots; dried peach; Other dried fruit; All types of Natural and Artificial fruit juices
Vegetables	Cucumber; Cabbage; Cauliflower; Brussels sprouts; Kale; Carrots (Row or boiled); Squash; Spinach; Lettuce; Mixed vegetables (Row or cocked) Eggplant; Celery; Kohlrabi; Green Peas; Green Beans; Turnip; Corn; Mushrooms; Onions; Beet; Beet root; Artichokes; Bell pepper; Garlic; Pepper
Legumes	Beans; Peas; Lima Beans; Broad Beans; Lentils; Soy; Split Peas; Mung beans;
Potatoes	Potatoes; French fries
Whole grains	Iranian bread (Sangak); Local bread (Tanoori); Wheat germ; Oatmeal; barley
Refined grains	White breads (lavash; barbari; baguettes); Noodles; Pasta; Rice
Pizza	Pizza
Condiments	Black pepper; Fried onion; Pomegranate paste; other sauces and pastes
Snacks	Potato chips; Corn puffs; biscuits and wafers
Nuts	Peanuts; Almonds; Pistachios; Hazelnuts; Walnuts; Sunflower; pumpkin and watermelon seeds
Mayonnaise	Mayonnaise sauce
Olive‐group	Olives; Olive oil
Vegetable oils	Vegetable oils (except for olive oil)
Sweets and Sugars	Chocolates; Cookies; Cakes; Confections; Traditional Sweets (Kamache sen; Poshtzik); Ardeh; Jam; Honey; Sugars; Candies; Syrup; Nabat (An Iranian confectionery made of sugar and served by tea); Noql (An Iranian confectioner (Liquid Sesame); Hlava Shekari (A Sweet Breakfast Food In Iran)
Soft drinks	Soft drinks
Tea & coffee	Tea; coffee; and espresso
Pickles	Pickles

**Table 2:** Factor loading matrix for the major dietary patterns identified among the sample of Iranian adults (*n* = 3,943) in Shahedieh cohort study (suburb area)

Food groups	Dietary patterns		
	Traditional	Unhealthy	Prudent
Vegetables	0.51	−0.24	0.51
Red & processed meats	0.48	—	—
Fish	0.46	—	—
Soft drinks	0.46	—	−0.33
Fruits	0.43	−0.23	0.51
Pickles	0.43	—	—
Nuts	0.43	—	—
Egg	0.41	0.30	—
Legumes	0.40	—	—
Dairy	0.40	−0.20	0.20
Mayonnaise	0.39	—	—
Potatoes	0.36	0.22	—
Refined grains	0.36	—	−0.21
Snacks	0.36	0.21	−0.36
Poultry	0.33	—	—
Vegetable oils	0.25	—	−0.20
Sweets and Sugars	0.22	0.54	—
Olive‐group	0.22	−0.23	—
Tea & coffee	0.21	0.50	—
Whole grains	—	—	0.26
Pizza	—	—	−0.42
% of variance explained	10.45	9.60	7.90

Note

Only items with correlation coefficients ≥.20 are presented.

In YaHS and TAMYZ study, 74.8% of the participants were in the age range of 20–49 years and 25.1% were above 50 years old. Prevalence of obesity was 21.2% (men, 8.6%; women, 12.6%). Two dietary patterns with 33.7% of the whole variance were recognized: “Traditional” (highly loaded by red and processed meats, vegetables, dairy, fruits, legumes, poultry, whole grains, fish, pickles, refined grains, eggs, and sweet and sugars), and "Unhealthy" (highly loaded by sweet and sugars, condiments, snack, soft drink, nuts, mayonnaise, tea, and coffee). Table 3 represents the factor loading matrixes of these dietary patterns. The participants' characteristics and dietary intakes across dietary patterns' quartiles are shown in Tables S2 and S4, respectively. In comparison with participants in the lowest quartile, members of the highest quartile of the traditional pattern were more likely to be male, younger and had a higher SES, and height but less likely to be centrally obese. Moreover, participants in the top quartile of the unhealthy pattern were more likely to be male, centrally obese but they had a lower SES. Consumption of sweet and sugar, condiment, snack, soft drink, tea and coffee, mayonnaise, and nuts intake across quartiles of unhealthy dietary pattern increased significantly; however, this relationship was inverse across quartiles of traditional dietary pattern. Moreover, energy intake increased significantly across quartiles of both dietary patterns.

**Table 3:** Factor loading matrix for the major dietary patterns identified among the sample of Iranian adults (*n* = 6,750) in YaHS cohort study (urban area)

Food groups	Dietary patterns	
	Traditional	Unhealthy
Red & processed meats	0.79	—
Vegetables	0.76	—
Dairy	0.75	—
Fruits	0.61	—
Legumes	0.61	—
Poultry	0.57	—
Whole grains	0.54	—
Fish	0.47	—
Pickles	0.45	—
Refined grains	0.27	—
Eggs	0.26	—
Sweets and Sugars	0.25	0.76
Condiments	—	0.68
Snacks	—	0.64
Nuts	—	0.61
Soft drinks	—	0.61
Mayonnaise	—	0.49
Tea & coffee	—	0.37
% of variance explained	19.4	14.3

Note

Only items with correlation coefficients ≥.20 are presented.

### 3.2 Dietary patterns and obesity in Shahedieh study (suburb area)

Table 4 represents the multivariable‐adjusted OR for obesity across dietary pattern scores' quartiles. In comparison with participants with the lowest adherence to “traditional” dietary pattern, those with highest adherence were significantly associated with greater odds of obesity after adjusting for the confounding variables such as age and energy intake (OR: 1.36, 95% CI: 1.11, 1.69), and also, further confounding variables such as gender, smoking and SES, marital status, physical activity levels, and chronic diseases (OR: 1.38, 95% CI: 1.11, 1.72). Moreover, these results remained significant in men when analysis was stratified by gender (OR: 1.49; 95% CI: 1.10, 2.00). Greater adherence to the "unhealthy" dietary pattern was associated with higher odds of overweight and obesity after confounders' adjustments (OR: 1.24; 95% CI: 1.02, 1.50); this result remained significant only for women (OR: 1.35; 95% CI: 1.01, 1.82). However, participants with the highest adherence to the "prudent" dietary pattern had significantly lower odds of obesity than those with the lowest adherence before (OR: 0.66; 95% CI: 0.52, 0.84) and after the full adjustments (OR: 0.68; 95% CI: 0.53, 0.58); this result remained significant for men (OR: 0.52; 95% CI: 0.37, 0.72). There was a significant increasing trend in the odds of obesity across increasing quartiles of the traditional dietary pattern in whole population before (*p*‐trend = .005) and after full adjustments (*p*‐trend = .04). Also, this result remained significant for males before (*p*‐trend = .008) and after full adjustments (*p*‐trend = .008). In addition, there was a significant increasing trend in the odds of obesity across increasing quartiles of the unhealthy dietary pattern among whole population after adjustment for age and energy intake (*p*‐trend = .01). This result remained significant among women (*p*‐trend = .001). However, there was a significant decreasing trend in the odds of obesity across increasing quartiles of the prudent dietary pattern among whole population before (*p*‐trend ˂ .001) and after full adjustments (*p*‐trend = .001). This result remained significant for men before (*p*‐trend ˂ .001) and after full adjustments (*p*‐trend ˂ .001).

**Table 4:** Odds ratio (95% CI) for obesity according to quartiles (Q) of dietary pattern in a sample of Iranian adults (*n* = 3,943) and also stratified by gender in Shahedieh cohort study (suburb area)^1^

	“Traditional” dietary pattern					“Unhealthy” dietary pattern					“Prudent” dietary pattern				
	Q1	Q2	Q3	Q4	*p*‐trend	Q1	Q2	Q3	Q4	*p* ternd	Q1	Q2	Q3	Q4	*p*‐trend
Whole papulation															
Model I^a^	1	1.01 (0.83–1.23)	1.01 (0.82–1.24)	1.36 (1.11–1.69)	**.005**	1	1.10 (0.91–1.32)	1.05 (0.87–1.27)	1.20 (0.99–1.45)	**.01**	1	0.92 (0.77–1.11)	0.75 (0.61–0.92)	0.66 (0.52–0.84)	**˂.001**
Model II^b^	1	1.01 (0.83–1.23)	1.01 (0.83–1.26)	1.38 (1.11–1.72)	**.04**	1	1.12 (0.92–1.36)	1.05 (0.86–1.27)	1.24 (1.02–1.50)	**.06**	1	0.91 (0.75–1.1)	0.75 (0.60–0.92)	0.68 (0.53–0.88)	**.001**
Men															
Model I	1	1.16 (0.85–1.56)	1.11 (0.83–1.5)	1.48 (1.11–1.98)	**.008**	1	1.03 (0.78–1.35)	1.17 (0.90–1.53)	1.20 (0.93–1.56)	**.10**	1	0.78 (0.59–1.03)	0.67 (0.5–0.89)	0.49 (0.35–0.67)	**˂.001**
Model II	1	1.13 (0.83–1.54)	1.08 (0.79–1.47)	1.49 (1.10–2.00)	**.008**	1	1.01 (0.76–1.34)	1.1 (0.84–1.46)	1.2 (0.91–1.57)	**.13**	1	0.78 (0.59–1.05)	0.68 (0.51–0.92)	0.52 (0.37–0.72)	**˂.001**
Women															
Model I	1	0.95 (0.73–1.23)	0.97 (0.73–1.3)	1.23 (0.89–1.7)	**.27**	1	1.18 (0.91–1.53)	0.93 (0.72–1.21)	1.27 (0.96–1.69)	**.001**	1	1.08 (0.83–1.39)	0.84 (0.62–1.13)	1.03 (0.71–1.49)	**.71**
Model II	1	0.96 (0.74–1.26)	1.01 (0.75–1.37)	1.27 (0.91–1.78)	**.19**	1	1.24 (0.95–1.62)	0.98 (0.75–1.29)	1.35 (1.01–1.82)	**.19**	1	1.02 (0.79–1.34)	0.80 (0.59–1.09)	1.001 (0.67–1.47)	**.56**

^1^ Data are OR (95% CI).

^a^ Model I: adjusted for age; and total energy intake.

^b^ Model II: in addition to age and total energy intake additionally adjusted for age; gender; smoking status; socioeconomic status; marital status; physical activity level; diseases and total energy intake.

Statistically significant *p*‐values (*p* ˂ .05) should be indicated in bold.

### 3.3 Dietary patterns and obesity in YaHS and TAMYZ study (urban area)

Table 5 represents the multivariable‐adjusted OR for obesity across dietary pattern scores' quartiles. Odds of obesity was higher in participants with the highest adherence to “unhealthy” dietary pattern compared to those with the lowest adherence after adjusting for the confounding variables such as age and energy intake (OR: 1.20, 95% CI: 1.01, 1.40) and also after further adjustments for gender, smoking and SES, marital status, physical activity levels, and chronic diseases (OR: 1.21, 95% CI: 1.02, 1.44). After gender stratification, the result remained significant in women (OR: 1.39, 95% CI: 1.07, 1.82). Although higher adherence to the "traditional" dietary pattern was not significantly associated with odds of obesity, but it was significantly associated with lower odds of obesity in women (OR: 0.74; 95% CI: 0.57, 0.95). In addition, there was a significant increasing trend in the odds of obesity across increasing quartiles of the unhealthy dietary pattern among whole population before (*p*‐trend = .02) and after full adjustments (*p*‐trend = .01). This result remained significant for women before (*p*‐trend = .002) and after full adjustments (*p*‐trend = .006).

**Table 5:** Odds ratio (95% CI) for obesity according to quartiles (Q) of dietary pattern in a sample of Iranian adults (*n* = 6,750); and also stratified by gender in YaHS cohort study (urban area)^1^

	“Traditional” dietary pattern					“unhealthy” dietary pattern				
	Q1	Q2	Q3	Q4	*p*‐trend	Q1	Q2	Q3	Q4	*p*‐trend
Whole population										
Model I^a^	1	0.92 (0.78–1.07)	0.94 (0.81–1.10)	1.07 (0.91–1.26)	.40	1	1.02 (0.88–1.19)	1.10 (0.94–1.28)	1.20 (1.01–1.40)	.02
Model II^b^	1	0.90 (0.76–1.06)	0.93 (0.79–1.10)	1.07 (0.91–1.27)	.38	1	1.04 (0.88–1.22)	1.12 (0.95–1.32)	1.21 (1.02–1.44)	.01
Men										
Model I	1	1.03 (0.83–1.26)	1.09 (0.89–1.34)	1.10 (0.89–1.36)	.24	1	1.05 (0.85–1.29)	1.01 (0.83–1.24)	1.05 (0.85–1.30)	.70
Model II	1	0.99 (0.79–1.23)	1.10 (0.88–1.37)	1.11 (0.88–1.39)	.28	1	1.09 (0.87–1.35)	1.07 (0.86–1.33)	1.08 (0.86–1.36)	.51
Women										
Model I	1	0.79 (0.62–1.02)	0.76 (0.60–0.96)	1.02 (0.79–1.31)	.87	1	1.00 (0.79–1.26)	1.22 (0.96–1.54)	1.40 (1.10–1.79)	.002
Model II	1	0.78 (0.61–1.01)	0.74 (0.57–0.95)	0.99 (0.76–1.29)	.98	1	1.00 (0.78–1.28)	1.20 (0.92–1.55)	1.39 (1.07–1.82)	.006

^1^ Data are OR (95% CI).

^a^ Model I: adjusted for age and energy intake.

^b^ Model II: in addition to age and total energy intake additionally adjusted for age; gender; smoking status; socioeconomic status; marital status; physical activity level; diseases and total energy intake.

## 4 DISCUSSION

According to the results, unhealthy dietary pattern increased the odds of obesity in residents of the suburb and urban areas. In addition, traditional dietary pattern increased the odds of obesity while the prudent dietary pattern was inversely related to obesity in suburb region. In men with greater adherence to the traditional dietary pattern, the odds of obesity increased. However, the odds of obesity were lower for higher adherence to the prudent dietary pattern in the suburb region. Moreover, the odds of obesity were higher in women with higher adherence to unhealthy pattern. In YaHS and TAMYZ studies, odds of obesity were lower in women with higher adherence to traditional pattern.

To the best of our knowledge, this was the first study conducted in the Middle East on the relationship between major dietary patterns derived from posteriori methods and obesity among a large sample of both urban and suburb regions.

Greater adherence to unhealthy dietary pattern was accompanied by higher odds of obesity in residents of both suburb and urban areas. In line with our results, other studies found that greater adherence to unhealthy/western dietary patterns were associated with more odds of obesity (Esmaillzadeh & Azadbakht, 2008; Mirzababaei et al., 2019; Rezazadeh & Rashidkhani, 2010; Shaker‐Hosseini & Ghodrati, 2017). Similar findings were reported from Canadian (Paradis et al., 2009), Argentinean (Pou et al., 2016), and Mexican adults (Denova‐Gutiérrez et al., 2011). In addition, a meta‐analysis including 13 articles from different countries of Asia, Europe, and the United States (Rezagholizadeh, Djafarian, Khosravi, & Shab‐Bidar, 2017) confirmed our results.

Unhealthy patterns contain high amounts of sweet and sugar and provide carbohydrate energy, so they differ from energy provided by fat (Hill & Prentice, 1995). High intake of carbohydrates leads to weight gain caused by consumption of high concentration of dietary fat with sugar, which increases the odds of obesity (Hill & Prentice, 1995). In unhealthy dietary pattern, consumption of snacks contributes to high caloric intake of saturated fat, cholesterol, and salt (Heald, 1992).

Greater adherence to prudent dietary pattern was associated with lower odds of obesity in the participants of suburb region. Prudent/healthy dietary patterns decreased the odds of obesity in a study over a small sample of Iranian women (Esmaillzadeh & Azadbakht, 2008; Mirzababaei et al., 2019; Rezazadeh & Rashidkhani, 2010). Studies from Canada, and Mexico, Argentina, and Mexico reported similar findings. These results were also confirmed in a meta‐analysis (Rezagholizadeh et al., 2017).

The odds of obesity are reduced in the prudent dietary pattern, since it is rich in fruits and vegetables, which contain fiber (Burton‐Freeman, 2000; Glore, Van Treeck, Knehans, & Guild, 1994; Zank & Kemp, 2012). In addition, chewing fiber requires more time than normal foods (Heaton, 1973) and it absorbs more water, creates a viscous gel that increases stomach distention (Howarth, Saltzman, & Roberts, 2001), provides low energy, and slows down the gastric emptying (Ruhee & Suzuki, 2018). Fiber also creates a feeling of satisfaction, decreases serum insulin secretion, and reduces the food intake (Ruhee & Suzuki, 2018). Fiber fermentation produces short‐chain fatty acids that modify the eating patterns by releasing the peptides and gut hormones, such as cholecystokinin and glucagon‐like peptide 1 (Anderson, Smith, & Gustafson, 1994; Du et al., 2009; Yao & Roberts, 2001). As a result, it reduces the individuals' hunger and promotes their satiety (Anderson et al., 1994; Du et al., 2009; Yao & Roberts, 2001). Phytochemicals present in fruits and vegetables act as prebiotics because of their nondigestible food components (e.g., fructo‐oligosaccharides) that modulate the gut microbiota (Schrezenmeir & de Vrese, 2001). As a result, it reduces the prevalence of obesity (Carrera‐Quintanar et al., 2018). The prudent dietary pattern is loaded with dairy products. Some studies showed useful effects of dairy products on weight loss (Beydoun et al., 2008; Snijder et al., 2007; Vergnaud et al., 2008) and decreased prevalence of central obesity (Azadbakht & Esmaillzadeh, 2008; Azadbakht, Mirmiran, Esmaillzadeh, & Azizi, 2005). The effect of dairy products on the absorption of fat (Jandacek, 1991), appetite, and metabolic activity of the gut microbiota (Marette & Picard‐Deland, 2014; Moreno, Bel‐Serrat, Santaliestra‐Pasías, & Bueno, 2015) is one of the underlying mechanisms affecting weight loss (Green, Stevenson, & Rumbold, 2017). Moreover, the literature showed that the prudent dietary pattern was inversely associated with unhealthy constituents such as snack and fast foods (Heald, 1992), soft drinks (Garduño‐Alanís et al., 2019; Katzmarzyk et al., 2016), and refined grains (Liu et al., 2003) that increased the odds of obesity.

Adherence to the traditional dietary pattern increased the odds of obesity. Our findings are consistent with the results of previous data collected from 141 Iranian adults. (Sherafat‐Kazemzadeh et al., 2010). This result was also in the same line with the research carried out among Chinese adults (Shu et al., 2015; Yu et al., 2015; Zhang et al., 2015), young Japanese women (Okubo et al., 2008), and the Mexican American population (Carrera, Gao, & Tucker, 2007). Conversely, in a case–control study including 147 Iranian adults, participants in the highest quartile of traditional dietary pattern had significantly lower odds of obesity (Yosaee et al., 2016). This result was also confirmed by a study on old (Xu, Byles, Shi, McElduff, & Hall, 2016) and young (Zhang et al., 2015) Chinese population. In the same vein, a cross‐sectional study over 486 women showed no significant association between Iranian dietary pattern (high in refined grains, potato, tea, whole grains, hydrogenated fats, legumes, and broth) and general obesity (Esmaillzadeh & Azadbakht, 2008). Similarly, no significant relationship was found between Lebanese traditional dietary pattern and obesity (Naja et al., 2011). The present discrepancies in the findings can be justified by referring to the observed variety in contents of traditional dietary patterns in different studies, interactions of these foods in the dietary pattern, and various confounder adjustment methods.

Increased odds of obesity by adherence to the traditional pattern can be explained by unhealthy components of this pattern. According to the literature, high consumption of red meat and its products increases the odds of obesity (Prentice & Jebb, 2003; Rouhani, Salehi‐Abargouei, Surkan, & Azadbakht, 2014), which is due to its high‐energy density (Xu, Yin, & Tong, 2007), cholesterol, and saturated fatty acids (Rouhani, Mirseifinezhad, Omrani, Esmaillzadeh, & Azadbakht, 2012). In Iranian eating habits, pickles, such as traditional appetizers, can be related to higher energy intake (Rouhani, Agh, & Azadbakht, 2018). In addition, soft drinks increase the odds of obesity, since they are full of sugar (Garduño‐Alanís et al., 2019; Katzmarzyk et al., 2016) and produce a lower sense of satiety (Pan & Hu, 2011). Refined grains, as the main portion of grains consumed by Iranian population (Bahadoran, Mirmiran, Delshad, & Azizi, 2014), are less nutrient‐dense, and have lower fiber (McKeown et al., 2010), so they increase odds of obesity (Liu et al., 2003).

Our results showed that traditional dietary pattern increased the obesity prevalence in Shahedieh (suburb) study, but not in YaHS (85% urban /15% suburb area) study. Considering that Shahedieh is a suburb region, its residents have different dietary quality and lifestyle than the urban region.

### 4.1 Strengths and limitations

Regarding this study's strengths, to the best of our knowledge, this was the first research on the relationship of dietary patterns with obesity in urban and suburb regions of Iran. Considering the variety of diet cultures and backgrounds, we aimed to identify the specific dietary patterns among adults in suburb and urban areas of Yazd City. Furthermore, administration of a validated semi‐quantitative FFQ to collect the study data by a face‐to‐face interview using trained interviewers ensured the data accuracy. Third, with regard to the reliability, a wide range of potential confounders were adjusted in this study.

Considering this study limitations, the following can be mentioned: in this cross‐sectional study, the causal relationship between dietary patterns and obesity could not be assessed. Consequently, further prospective studies are required in this area. Second, generalizability of the dietary patterns to the whole population of Iran is not possible, since dietary intakes in Yazd are different from other cities. In addition, the researchers had to make several subjective decisions to conduct this study; for example, categorizing food items into food groups, extracting the number of factors, applying the rotation method, and labeling the factors. Moreover, we cannot reject the possibility of residual confounding bias, since unknown or unmeasured confounders may exist that affected our results. Finally, our participants with odds of obesity might have been advised to reduce their fat intake, which led them to alter their dietary habits. However, such possibility cannot be resolved in a cross‐sectional study.

## 5 CONCLUSION

Greater adherence to unhealthy dietary patterns was associated with higher odds of obesity in residents of suburb and urban areas in Yazd Greater Area‐Iran. In suburb area, greater adherence to traditional dietary pattern was associated with higher odds of obesity, while adherence to prudent dietary pattern was associated with lower odds of obesity. In order to reflect on the causal relationship between the studies variables, further prospective studies are needed.

## CONFLICT OF INTEREST

The authors declare no conflict of interest to report regarding this study.

## AUTHOR CONTRIBUTION

SS‐KH and MH made substantial contributions to the conception and design of the manuscript, preparation manuscript, as well as performing statistical analysis and data interpretation. They also approved the final manuscript for submission and critical revision. HM‐KH, MM, and AN contributed to data interpretation and also critically revised the manuscript for important intellectual content and approved the final manuscript for submission.

## ETHICAL APPROVAL

The study's protocols and procedures were ethically reviewed and approved by a recognized ethical body (Ethics Committee of Shahid Sadoughi University of Medical Science with ethics code of (IR.SSU.SPH.REC.1397.123)). This study does not involve any human or animal testing. Also, this study conforms to the Declaration of Helsinki, US, and/or European Medicines Agency Guidelines for human subjects.

## INFORMED CONSENT

Written informed consent was obtained from all study participants.

## Supporting information

Table S1
Table S2
Table S3
Table S4
