# Supplementary material for: Empirically derived dietary patterns and obesity among Iranian Adults: Yazd Health Study‐TAMYZ and Shahedieh cohort study
Source: Food Sci Nutr. 2020 Mar 31;8(5):2478–89. doi: 10.1002/fsn3.1538 (PMC7215230; doi:10.1002/fsn3.1538)
Supplement: Supplementary file 1 — Table S1 Table S2 Table S3 Table S4 [file FSN3-8-2478-s001.docx]

**SUPPLEMENTARY TABLE 1.** Participant characteristics and dietary intakes by quartile (Q) categories of dietary pattern scores in a sample of Iranian adults (n= 3943) in shahedieh cohort study (suburb area)^1^

| “Traditional” dietary pattern | | | |  |  | “Unhealthy” dietary pattern | | | | | “Prudent” dietary pattern | | | | |
| --- | --- | --- | --- | --- | --- | --- | --- | --- | --- | --- | --- | --- | --- | --- | --- |
|  | **Q1** | **Q2** | **Q3** | **Q4** | **^*^P** | **Q1** | **Q2** | **Q3** | **Q4** | **^*^P** | **Q1** | **Q2** | **Q3** | **Q4** | **^*^P** |
| Age (%) |  |  |  |  | **0.01** |  |  |  |  | **0.01** |  |  |  |  | **0.001** |
| 35-49 | 19.6 | 27.2 | 26.4 | 26.9 |  | 26.6 | 26.2 | 22.9 | 24.3 |  | 22.9 | 26.0 | 25.0 | 26.2 |  |
| 50-69 | 50.3 | 28.3 | 18.7 | 12.6 |  | 20.9 | 22.6 | 27.9 | 28.6 |  | 26.5 | 23.3 | 24.9 | 25.3 |  |
| Gender (female) (%) | 31.8 | 30.8 | 23.1 | 14.3 | **0.001** | 29.2 | 26.9 | 24.3 | 19.6 | **0.001** | 32.2 | 27.3 | 23.4 | 17.0 | **0.001** |
| Height (cm) | 161.2 ± 9.0 | 162.6 ± 9.0 | 165.5 ± 9.7 | 170.8 ± 52.3 | **0.001** | 162.9 ± 9.5 | 165.6 ± 52.7 | 165.2 ± 10.1 | 166.5 ± 9.2 | **0.02** | 164.5 ± 52.6 | 164.0 ± 9.9 | 165.1 ± 9.7 | 166.7 ± 9.5 | **0.14** |
| Weight (kg) | 71.8 ± 12.9 | 72.2 ± 13.3 | 74.4 ± 13.9 | 78.1 ± 14.4 | **0.001** | 71.8 ± 13.5 | 73.6 ± 13.7 | 74.6 ± 14.1 | 76.6 ± 13.6 | **0.001** | 73.7 ± 13.5 | 74.2 ± 13.9 | 74.2 ± 13.9 | 74.5 ± 14.0 | **0.60** |
| BMI (kg/m^2^) | 27.6 ± 4.7 | 27.3 ± 4.6 | 27.1 ± 4.4 | 27.2 ± 4. | **0.06** | 27.1 ± 4.8 | 27.3 ± 4.6 | 27.2 ± 4.5 | 27.6 ± 4.4 | **0.08** | 27.7 ± 4.4 | 27.5 ± 4.6 | 27.2 ± 4.5 | 26.8 ± 4.6 | **0.001** |
| Central obesity^2^ (%) | 29.5 | 27.3 | 22.8 | 20.3 | **0.001** | 26.2 | 26.1 | 25.3 | 22.4 | **0.01** | 27.9 | 26.9 | 24.4 | 20.8 | **0.001** |
| General obesity^3^ (%) | 27.9 | 25.1 | 22.8 | 24.1 | **0.004** | 25.0 | 25.1 | 23.8 | 26.1 | **0.66** | 26.4 | 26.9 | 23.2 | 23.5 | **0.10** |
| SES (%) |  | | |  | **0.20** |  | | | | **0.001** |  | | | | **0.01** |
| Weak | 26.5 | 25.3 | 23.6 | 24.7 |  | 32.8 | 25.3 | 21.4 | 20.6 |  | 22.7 | 24.9 | 24.7 | 27.7 |  |
| Middle | 22.3 | 24.6 | 24.6 | 24.8 |  | 24.0 | 26.0 | 25.0 | 25.0 |  | 24.5 | 24.9 | 26.4 | 24.2 |  |
| High | 25.0 | 25.1 | 24.9 | 25.0 |  | 16.4 | 23.7 | 29.4 | 30.5 |  | 27.9 | 24.3 | 24.2 | 22.6 |  |
| Marital status (married) (%) | 24.6 | 25.0 | 25.2 | 25.3 | **0.001** | 24.5 | 25.2 | 25.1 | 25.2 | **0.005** | 24.7 | 24.9 | 25.3 | 25.2 | **0.03** |
| Physical activity (≥1 h/week) (%) | 26.4 | 25.6 | 26.2 | 26.1 | **0.06** | 24.2 | 24.3 | 27.5 | 23.9 | **0.001** | 26.0 | 26.5 | 25.3 | 22.2 | **0.07** |
| Current smoking (%) | 16.6 | 18.4 | 25.9 | 39 | **0.07** | 23.2 | 24.3 | 25.0 | 25.5 | **0.21** | 20.5 | 20.4 | 18.9 | 18.2 | **0.19** |

^1^ Categorical variables are presented as sum and percentages; and continuous variables are presented as Mean ± standard deviation (SD). Quartiles of dietary pattern score are presented by Q1; Q2; Q3; Q4; n = 3943. Body mass index; BMI; Socioeconomic status; SES.

^2^ Central obesity: waist circumference ≥ 88 cm.

^3^ General obesity: BMI ≥ 30 kg/m^2^.

^*^ P values for continuous variables (analysis of variance) and for categorical variables (chi-square test). P < 0.05 was considered statistically significant.

**SUPPLEMENTARY TABLE** **2.** Participant characteristics and dietary intakes by quartile (Q) categories of dietary pattern scores in a sample of Iranian adults (n = 6750) in YaHS cohort study (urban area)^1^

| “Traditional” dietary pattern | | | | | | | “Unhealthy” dietary pattern | | | | |
| --- | --- | --- | --- | --- | --- | --- | --- | --- | --- | --- | --- |
|  | **Q1** | | **Q2** | **Q3** | **Q4** | **^*^P** | **Q1** | **Q2** | **Q3** | **Q4** | **^*^P** |
| Age (%) |  | |  |  |  | **0.007** |  |  |  |  | **0.46** |
| 20-49 | 24.5 | | 26.4 | 22.9 | 26.2 |  | 24.0 | 25.1 | 26.4 | 24.4 |  |
| 50-70 | 28.0 | | 28.9 | 22.9 | 20.2 |  | 27.0 | 27.2 | 21.9 | 23.9 |  |
| Gender (female) (%) | | 25.7 | 26.4 | 24.7 | 23.2 | **0.02** | 25.4 | 25.5 | 24.7 | 24.4 | **0.001** |
| Height (cm) | 164.9 ± 10.5 | | 164.9 ± 10.0 | 165.7 ± 9.9 | 166.2 ± 10.3 | **0.002** | 165.1 ± 10.2 | 165.4 ± 10.1 | 165.6 ± 10.2 | 166.7 ± 10.4 | **0.37** |
| Weight (kg) | 71.7 ± 14.7 | | 71.4 ± 14.4 | 71.7 ± 14.9 | 72.8 ± 14.5 | **0.43** | 71.6 ± 14.9 | 71.8 ± 14.9 | 71.9 ± 14.4 | 72.4 ± 14.4 | **0.49** |
| BMI (kg/m^2^) | 26.3 ± 4.9 | | 26.3 ± 4.9 | 26.3 ± 5.1 | 26.0 ± 4.8 | **0.28** | 26.3 ± 4.9 | 26.2 ± 5.0 | 26.2 ± 5.0 | 26.3 ± 4.7 | **0.87** |
| Central obesity^2^ (%) | 26.3 | | 25.5 | 24.8 | 23.4 | **0.001** | 24.7 | 24.3 | 24.8 | 26.2 | **0.02** |
| General obesity^3^ (%) | 25.1 | | 24.4 | 23.5 | 27.0 | **0.20** | 24.7 | 25.5 | 24.8 | 24.9 | **0.96** |
| SES (%) |  | | | |  | **0.001** |  | | | | **0.009** |
| Low | 26.8 | | 24.5 | 25.0 | 23.6 |  | 25.0 | 23.9 | 24.9 | 26.2 |  |
| Middle | 25.6 | | 25.7 | 24.9 | 23.8 |  | 23.1 | 26.0 | 25.9 | 25.0 |  |
| High | 21.2 | | 24.2 | 24.4 | 30.2 |  | 28.6 | 24.3 | 22.7 | 24.4 |  |
| Marital status (married) (%) | 25.2 | | 25.4 | 25.0 | 24.4 | **0.053** | 24.9 | 24.6 | 25.3 | 25.2 | **0.69** |
| Physical activity (≥1 h/week) (%) | 25.0 | | 24.7 | 25.3 | 25.0 | **0.68** | 24.9 | 25.1 | 25.2 | 24.8 | **0.55** |
| Current smoking (%) | 26.9 | | 25.4 | 21.5 | 26.2 | **0.16** | 25.1 | 23.0 | 25.6 | 26.3 | **0.52** |

^1^ Categorical variables are presented as sum and percentages; and continuous variables are presented as Mean ± standard deviation (SD). Quartiles of dietary pattern score are presented by Q1; Q2; Q3; Q4; n = 6750. Body mass index; BMI; Socioeconomic status; SES.

^2^ Central obesity: waist circumference ≥ 88 cm.

^3^ General obesity: BMI ≥ 30 kg/m^2^.

^*^ P values for continuous variables (analysis of variance) and for categorical variables (chi-square test). P < 0.05 was considered statistically significant.

**SUPPLEMENTARY TABLE** **3.** Dietary intakes by quartile (Q) categories of dietary pattern scores in a sample of Iranian adults (n= 3943) in shahedieh cohort study (suburb area)^1^

| “Traditional” dietary pattern | | | | | | | |  | |  | | “Unhealthy” dietary pattern | | | | | | | | | | “Prudent” dietary pattern | | | | | | | | | |
| --- | --- | --- | --- | --- | --- | --- | --- | --- | --- | --- | --- | --- | --- | --- | --- | --- | --- | --- | --- | --- | --- | --- | --- | --- | --- | --- | --- | --- | --- | --- | --- |
|  | | **Q1** | | **Q2** | | **Q3** | | **Q4** | | **P** | | **Q1** | | **Q2** | | **Q3** | | **Q4** | | **P** | | **Q1** | | **Q2** | | **Q3** | | **Q4** | | **P** | |
| Red & Processed meats | | 29.2 ± 19.7 | | 36.9 ± 22.0 | | 46.1 ± 27.0 | | 62.5 ± 41.6 | | **˂ 0.001** | | 34.4 ± 25.2 | | 39.9 ± 25.7 | | 44.8 ± 28.3 | | 55.5 ± 40.4 | | **˂ 0.001** | | 37.9 ± 26.7 | | 42.1 ± 31.8 | | 44.1 ± 27.1 | | 50.6 ± 37.8 | | **˂ 0.001** | |
| Fish | | 2.6 ± 3.3 | | 4.1 ± 4.5 | | 5.9 ± 5.6 | | 10.3 ± 10.8 | | **˂ 0.001** | | 3.4 ± 4.6 | | 4.8 ± 4.7 | | 6.1 ± 6.8 | | 8.7 ± 10.3 | | **˂ 0.001** | | 6.4 ± 8.3 | | 5.6 ± 6.8 | | 5.7 ± 6.9 | | 5.3 ± 6.9 | | **0.08** | |
| Poultry | | 9.7 ± 8.3 | | 12.0 ± 9.6 | | 13.9 ± 12.0 | | 19.2 ± 19.0 | | **˂ 0.001** | | 10.0 ± 8.5 | | 12.2 ± 10.5 | | 14.7 ± 13.1 | | 18.0 ± 18.1 | | **0.02** | | 15.2 ± 16.9 | | 13.7 ± 13.0 | | 12.5 ± 10.7 | | 13.4 ± 11.9 | | **˂ 0.001** | |
| Eggs | | 26.2 ± 12.9 | | 26.2 ± 18.8 | | 33.0 ± 22.4 | | 42.7 ± 34.3 | | **˂ 0.001** | | 29.4 ± 24.6 | | 28.6 ± 21.2 | | 30.7 ± 22.8 | | 34.5 ± 31.0 | | **˂ 0.001** | | 19.7 ± 14.4 | | 27.2 ± 18.9 | | 33.1 ± 21.4 | | 43.3 ± 35.2 | | **˂ 0.001** | |
| Dairy products | | 116.7 ± 135.6 | | 124.1 ±131.2 | | 132.8 ± 135.1 | | 165.9 ± 163.9 | | **˂ 0.001** | | 66.6 ± 53.7 | | 102.3 ± 79.9 | | 135.2 ± 106.3 | | 235.4 ± 213.8 | | **˂ 0.001** | | 142.8 ± 162.2 | | 129.1 ± 128.6 | | 130.5 ± 128.7 | | 137.2 ± 150.3 | | **0.11** | |
| Fruits | | 418.8 ± 482.2 | | 425.4 ± 288.1 | | 426.5 ± 288.0 | | 490.0 ± 330.9 | | **˂ 0.001** | | 234.0 ± 133.5 | | 364.4 ± 160.5 | | 482.0 ± 200.6 | | 790.1 ± 512.0 | | **˂ 0.001** | | 450.5 ± 309.2 | | 446.5 ± 297.7 | | 447.4 ± 311.8 | | 526.3 ± 497.2 | | **˂ 0.001** | |
| Vegetables | | 205.9 ± 116.4 | | 205.9 ± 116.4 | | 215.3 ± 129.5 | | 223.5 ± 136.4 | | **˂ 0.001** | | 127.1 ± 51.4 | | 179.1 ± 59.6 | | 231.3 ± 76.5 | | 356.6 ± 182.7 | | **˂ 0.001** | | 211.3 ± 48.9 | | 218.3 ± 117.5 | | 220.8 ± 121.6 | | 243.6 ± 149.5 | | **˂ 0.001** | |
| Legumes | | 31.9 ± 30.4 | | 31.7 ±22.4 | | 36.1 ± 28.7 | | 43.9 ± 36.1 | | **˂ 0.001** | | 25.5 ± 17.1 | | 31.4 ± 21.7 | | 38.1 ± 30.5 | | 48.5 ± 40.9 | | **˂ 0.001** | | 24.9 ± 18.4 | | 32.5 ± 22.6 | | 37.5 ± 27.2 | | 49.1 ± 41.9 | | **˂ 0.001** | |
| Potatoes | | 33.6 ± 33.6 | | 37.4 ± 34.3 | | 39.5 ± 33.7 | | 46.0 ± 39.2 | | **˂ 0.001** | | 27.8 ± 22.4 | | 34.8 ± 28.3 | | 42.6 ± 35.1 | | 51.4 ± 47.1 | | **˂ 0.001** | | 24.0 ± 18.1 | | 33.3 ± 23.6 | | 40.5 ± 29.6 | | 58.8 ± 51.5 | | **˂ 0.001** | |
| Whole grains | | 122.1 ± 185.7 | | 206.1 ± 216.6 | | 270.6 ± 286.1 | | 317.0 ± 290.4 | | **˂ 0.001** | | 230.3 ± 282.6 | | 215.0 ± 249.6 | | 227.4 ± 251.7 | | 243.2 ± 251.4 | | **0.11** | | 198.3 ± 151.4 | | 256.5 ± 260.8 | | 233.9 ± 284.6 | | 227.2 ± 295.2 | | **˂ 0.001** | |
| Refined grains | | 171.8 ± 100.5 | | 222.4 ± 108.7 | | 261.6 ± 131.7 | | 329.3 ± 219.2 | | **˂ 0.001** | | 224.8 ± 139.2 | | 239.4 ± 165.6 | | 252.5 ± 144.4 | | 268.3 ± 178.7 | | **˂ 0.001** | | 232.6 ± 137.0 | | 244.3 ± 175.8 | | 245.9 ± 139.1 | | 262.3 ± 176.4 | | **0.01** | |
| Pizza | | 1.3 ±2.4 | | 2.9 ± 3.8 | | 5.3 ± 5.5 | | 13.7 ± 18.2 | | **˂ 0.001** | | 5.8 ± 12.9 | | 5.3 ± 8.7 | | 5.7 ± 10.1 | | 6.3 ± 11.2 | | **0.21** | | 6.5 ± 12.2 | | 5.9 ± 11.4 | | 5.5 ± 8.5 | | 5.3 ± 10.9 | | **0.06** | |
| Snacks | | 0.18 ± 0.56 | | 0.35 ± 0.74 | | 0.78 ± 1.37 | | 3.2 ± 6.5 | | **˂ 0.001** | | 1.3 ± 5.1 | | 0.9 ± 2.2 | | 1.0 ± 2.5 | | 1.26 ± 3.7 | | **0.01** | | 1.2 ± 3.8 | | 1.01 ± 3.4 | | 1.1 ± 3.3 | | 1.1 ± 3.6 | | **0.64** | |
| Nuts | | 10.5 ± 14.9 | | 14.2 ± 19.0 | | 19.6 ± 27.5 | | 37.8 ± 57.1 | | **˂ 0.001** | | 11.8 ± 21.3 | | 17.5 ± 34.7 | | 21.4 ± 35.0 | | 31.4 ± 44.1 | | **˂ 0.001** | | 14.0 ± 19.0 | | 17.3 ± 27.6 | | 19.6 ± 30.5 | | 31.3 ± 53.0 | | **0.01** | |
| Mayonnaise | | 0.53 ± 1.0 | | 0.93 ± 1.5 | | 1.4 ± 2.3 | | 3.5 ± 6.3 | | **˂ 0.001** | | 1.0 ± 2.0 | | 1.29 ± 2.4 | | 1.6 ± 2.5 | | 2.5 ± 6.0 | | **˂ 0.001** | | 1.3 ± 3.0 | | 1.6 ± 4.1 | | 1.6 ± 2.9 | | 1.8 ± 4.4 | | **0.005** | |
| Olive | | 2.1 ± 4.9 | | 2.3 ± 6.1 | | 2.6 ± 6.6 | | 3.3 ± 7.3 | | **˂ 0.001** | | 0.84 ± 2.3 | | 1.7 ± 3.6 | | 2.4 ± 4.3 | | 5.5 ± 10.5 | | **˂ 0.001** | | 4.0 ± 9.5 | | 2.4 ± 4.9 | | 2.0 ± 4.7 | | 2.0 ± 4.5 | | **0.01** | |
| Vegetable oils | | 7.2 ± 7.5 | | 10.5 ± 9.3 | | 14.1 ± 12.0 | | 19.3 ± 16.2 | | **˂ 0.001** | | 11.9 ± 12.2 | | 12.2 ± 11.6 | | 12.9 ± 12.1 | | 14.1 ± 14.2 | | **˂ 0.001** | | 14.4 ± 13.4 | | 12.4 ± 12.1 | | 12.2 ± 12.0 | | 12.1 ± 12.6 | | **0.01** | |
| Sweets & Sugars | | 54.6 ± 66.8 | | 46.4 ±57.2 | | 49.6 ± 60.3 | | 58.3 ± 74.4 | | **˂ 0.001** | | 63.2 ± 79.2 | | 52.0 ± 63.7 | | 46.6 ± 56.1 | | 46.6 ± 57.7 | | **˂ 0.001** | | 11.0 ± 19.8 | | 28.5 ± 27.8 | | 52.8 ± 37.0 | | 116.1 ± 90.1 | | **˂ 0.001** | |
| Soft drinks | | 24.1 ± 45.7 | | 38.6 ± 56.5 | | 64.1 ± 80.8 | | 198.6 ± 259.2 | | **˂ 0.001** | | 77.6 ± 161.5 | | 74.7 ± 169.3 | | 79.6 ± 141.9 | | 93.5 ± 151.9 | | **0.03** | | 65.8 ± 137.2 | | 71.4 ± 151.3 | | 78.9 ± 142.9 | | 109.2 ± 186.8 | | **˂ 0.001** | |
| Tea & coffee | | 723.3 ± 679.1 | | 679.3 ± 669.2 | | 683.7 ± 571.0 | | 789.5 ± 864.8 | | **˂ 0.001** | | 821.0 ± 807.0 | | 726.6 ± 739.9 | | 633.6 ± 566.3 | | 694.7 ± 671.9 | | **˂ 0.001** | | 380.5 ± 257.0 | | 545.2 ± 330.0 | | 692.0 ± 434.1 | | 1258.1 ± 1091.3 | | **˂ 0.001** | |
| Pickles | | 10.7 ± 17.5 | | 12.5 ± 18.2 | | 14.6 ± 23.0 | | 24.2 ± 32.0 | | **˂ 0.001** | | 6.4 ± 8.2 | | 10.7 ± 12.8 | | 15.5 ± 17.8 | | 29.5 ± 38.5 | | **˂ 0.001** | | 15.4 ± 26.6 | | 15.4 ± 25.2 | | 13.7 ± 17.6 | | 17.5 ± 25.2 | | **0.006** | |
| Energy intake (kcal/d) | 2227.6 ± 1883.7 | | 2430.1 ± 1534.9 | | 2550.1 ± 1344.4 | | 3280.7 ±1345.8 | | **˂ 0.001** | | 2483..1 ± 1883 | | 2612.3 ± 1549.4 | | 2921.2 ± 1532.2 | | 3371.7 ± 1516.7 | | **˂ 0.001** | | 1580.7 ± 875.3 | | 2323.7 ±1105.5 | | 3207.6 ± 1326.4 | | 4276 ± 1496.9 | | **˂ 0.001** | |  |

^1^ Variables are presented as Mean ± standard deviation (SD). Quartiles of dietary pattern score are presented by Q1; Q2; Q3; Q4; n = 3943.

^*^ P < 0.05 for continuous variables (analysis of variance) was considered statistically significant.

**SUPPLEMENTARY TABLE** **4.**  Dietary intakes by quartile (Q) categories of dietary pattern scores in a sample of Iranian adults (n= 6750) in YaHS (urban area)^1^

| “Traditional” dietary pattern | | | |  |  | “Unhealthy” dietary pattern | | | | |
| --- | --- | --- | --- | --- | --- | --- | --- | --- | --- | --- |
|  | **Q1** | **Q2** | **Q3** | **Q4** | **P** | **Q1** | **Q2** | **Q3** | **Q4** | **P** |
| Red & Processed meats | 61.3 ± 48.2 | 67.1 ± 43.9 | 100.4 ± 73.8 | 254.4 ± 448.6 | **˂ 0.001** | 134.8 ± 335.5 | 87.6 ± 89.0 | 112.6 ± 164.1 | 148.2 ± 293.5 | **˂ 0.001** |
| Fish | 12.3 ± 18.4 | 12.9 ± 23.3 | 19.3 ± 36.7 | 85.8 ± 283.6 | **˂ 0.001** | 50.5 ± 261.4 | 19.5 ± 44.4 | 24.1 ± 56.9 | 36.2 ± 111.4 | **˂ 0.001** |
| Poultry | 26.7 ± 29.7 | 42.5 ± 39.6 | 60.3 ± 65.8 | 159.9 ± 427.8 | **˂ 0.001** | 97.5 ± 408.2 | 59.3 ± 92.1 | 61.7 ± 78.1 | 70.9 ± 239.9 | **0.02** |
| Eggs | 16.1 ± 19.3 | 24.3 ± 20.0 | 31.5 ± 33.4 | 59.8 ± 162.9 | **˂ 0.001** | 46.4 ± 156.6 | 29.0 ± 39.2 | 29.6 ± 34.3 | 26.6 ± 45.0 | **˂ 0.001** |
| Dairy | 213.0 ± 153.2 | 268.5 ± 152.0 | 358.3 ± 250.8 | 757.0 ± 1717.9 | **˂ 0.001** | 437.7 ± 1331.8 | 330.4 ± 400.0 | 346.4 ± 313.7 | 482.4 ± 1093.3 | **˂ 0.001** |
| Fruits | 352.6 ± 250.9 | 471.8 ± 297.7 | 679.7 ± 522.7 | 1418.9 ± 2303.7 | **˂ 0.001** | 594.7 ± 583.7 | 600.7 ± 714.1 | 1003.9 ± 2134.0 | 730.8 ± 1266.5 | **˂ 0.001** |
| Vegetables | 120.2 ± 103.2 | 158.9 ± 91.8 | 224.3 ± 145.2 | 556.9 ± 940.0 | **˂ 0.001** | 228.5 ± 476.5 | 217.2 ± 260.5 | 263.0 ± 325.8 | 351.7 ± 794.3 | **˂ 0.001** |
| Legumes | 29.3 ± 22.9 | 35.2 ± 25.6 | 49.6 ± 39.8 | 101.4 ± 181.5 | **˂ 0.001** | 48.1 ± 44.0 | 42.8 ± 48.7 | 53.7 ± 69.9 | 70.7 ± 135.0 | **˂ 0.001** |
| Potatoes | 5.1 ± 7.1 | 7.7 ± 9.4 | 12.6 ± 20.5 | 25.0 ± 58.5 | **˂ 0.001** | 13.3 ± 40.4 | 13.4 ± 23.0 | 12.7 ± 25.8 | 11.12 ± 37.0 | **0.17** |
| Whole grains | 38.7 ± 39.0 | 70.3 ± 49.3 | 84.0 ± 67.9 | 134.8 ± 211.6 | **˂ 0.001** | 83.8 ± 108.7 | 80.4 ± 75.5 | 88.3 ± 98.3 | 75.3 ± 175.7 | **0.02** |
| Refined grains | 147.5 ± 139.0 | 212.6 ± 127.4 | 248.2 ± 185.7 | 409.0 ± 704.6 | **˂ 0.001** | 250.5 ± 227.3 | 242.1 ± 215.2 | 275.4 ± 408.1 | 249.4 ± 559.7 | **0.09** |
| condiment | 44.5 ±118.1 | 22.6 ± 44.5 | 28.2 ± 49.9 | 37.3 ± 70.2 | **˂ 0.001** | 11.6 ± 10.7 | 17.0 ± 13.0 | 25.4 ± 20.9 | 78.2 ± 141.7 | **0.21** |
| Snacks | 40.7 ± 110.8 | 13.4 ± 34.1 | 15.9 ± 44.5 | 21.5 ± 69.6 | **˂ 0.001** | 5.8 ± 10.9 | 7.6 ± 9.4 | 11.3 ± 15.8 | 66.7 ± 133.0 | **˂ 0.001** |
| Nuts | 43.1 ± 89.3 | 22.4 ± 52.2 | 24.9 ± 41.1 | 36.5 ± 83.8 | **˂ 0.001** | 9.7 ± 10.0 | 13.4 ± 13.1 | 22.8 ± 22.1 | 81.0 ± 125.2 | **˂ 0.001** |
| Mayonnaise | 3.8 ± 10.7 | 2.7 ± 3.5 | 2.1 ± 4.2 | 2.0 ± 5.0 | **˂ 0.001** | 0.79 ± 1.0 | 1.1 ± 1.3 | 2.0 ± 2.9 | 6.6 ± 12.8 | **˂ 0.001** |
| Sweets & Sugars | 267.9 ± 491.9 | 116.1 ± 203.1 | 146.5 ± 223.6 | 251.4 ± 382.0 | **˂ 0.001** | 80.93 ± 85.34 | 91.1 ± 82.8 | 125.4 ± 117.7 | 484.5 ± 596.3 | **˂ 0.001** |
| Soft drinks | 529.8 ± 1549.6 | 137.9 ± 391.4 | 174.3 ± 518.3 | 233.3 ± 684.8 | **˂ 0.001** | 48.7 ± 84.0 | 73.5 ± 104.7 | 124.0 ± 210.0 | 828.8 ± 1689.5 | **0.03** |
| Tea & coffee | 539.6 ± 1060.8 | 460.9 ± 442.7 | 422.3 ± 565.6 | 368.7 ± 610.2 | **˂ 0.001** | 210.0 ± 224.1 | 378.1 ± 281.8 | 520.0 ± 428.3 | 683.0 ± 1262.3 | **˂ 0.001** |
| Pickles | 9.2 ± 10.4 | 11.0 ± 10.3 | 20.5 ± 27.7 | 49.7 ± 102.5 | **˂ 0.001** | 18.9 ± 44.0 | 18.4 ± 38.7 | 20.7 ± 41.0 | 32.2 ± 56.0 | **˂ 0.001** |
| Energy intake (kcal/d) | 2991.0 ± 3019.7 | 2379.8 ± 1298.4 | 3072.4 ± 1424.5 | 5980.8 ±6594.8 | **˂ 0.001** | 2886.2 ± 3572.5 | 2515.4 ± 1392.3 | 3114.9 ± 1973.3 | 5907.5 ± 6188.1 | **˂ 0.001** |

^1^ Variables are presented as Mean ± standard deviation (SD). Quartiles of dietary pattern score are presented by Q1; Q2; Q3; Q4; n = 6750.

^*^ P < 0.05 for continuous variables (analysis of variance) was considered statistically significant.
